# Supplementary material for: Separating selection from mutation in antibody language models
Source: bioRxiv. 2025 Oct 22:2025.10.21.683652. Preprint. [Version 1] doi: 10.1101/2025.10.21.683652 (PMC12633574; doi:10.1101/2025.10.21.683652)
Supplement: 1 [file NIHPP2025.10.21.683652V1-supplement-1.pdf]

bioRxiv preprint doi: https://doi.org/10.1101/2025.10.21.683652; this version posted October 22, 2025. The copyright holder for this preprint (which was not certified by peer review) is the author/funder, who has granted bioRxiv a license to display the preprint in perpetuity. It is made available under aCC-BY 4.0 International license.

896 # Supplementary Materials

purpose	name	samples	clonal families	PCPs	median mutations
train	`JaffePairedCC`	4	50,776	209,599	7
train	`TangCC`	21	45,267	651,899	2
train	`VanwinkleheavyTrainCC`	149	21,269	124,985	4
train	`VanwinklelightTrainCC1m`	330	2,658	1,000,000	2
test	`RodriguezCC`	51	3,592	38,050	5

Table S1: Data used in this paper. CC means that PCPs with mutated cysteines are excluded from the data. `JaffePairedCC` is paired data from [56] sequenced using 10X. `TangCC` data is heavy chain data from [55, 57] and was sequenced using the methods of [57]. `VanwinkleheavyTrainCC1m` is a subset of the heavy chain data from [58] sequenced using Takara 5'RACE BCR kit. `VanwinklelightTrainCC1m` is a 1M subset of the light chain data from [58] sequenced using Takara 5'RACE BCR kit. `RodriguezCC` data is the 5′ RACE heavy chain data from [12] and is used only for testing. The "samples" column is the number of individual samples in the dataset; in these datasets, each sample is from a distinct individual. "Clonal families" is the number of clonal families in the dataset. "PCPs" is the number of parent-child pairs in the dataset. "Median mutations" is the median number of mutations per PCP in the dataset.

bioRxiv preprint doi: https://doi.org/10.1101/2025.10.21.683652; this version posted October 22, 2025. The copyright holder for this preprint (which was not certified by peer review) is the author/funder, who has granted bioRxiv a license to display the preprint in perpetuity. It is made available under aCC-BY 4.0 International license.

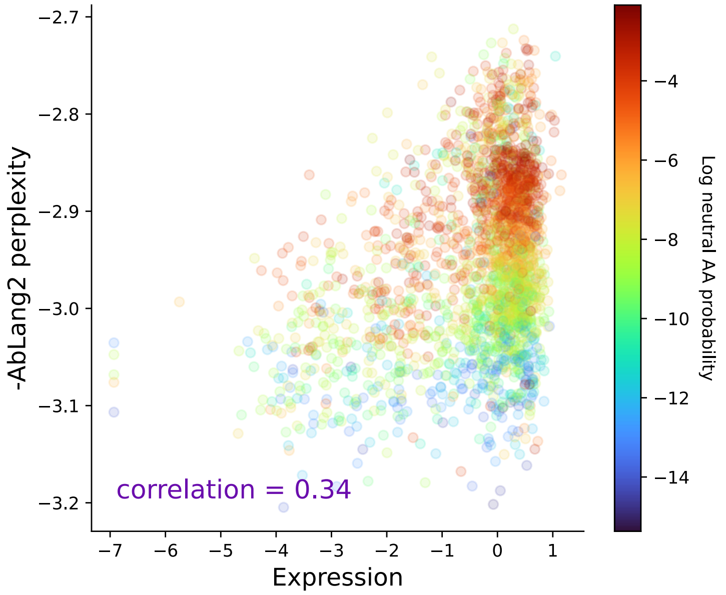

Figure S1: Conflating mutation and selection hinders functional prediction. This plot is analogous to the rightmost panel of Figure 1c, but colored by mutability according to the Thrifty [7] model.

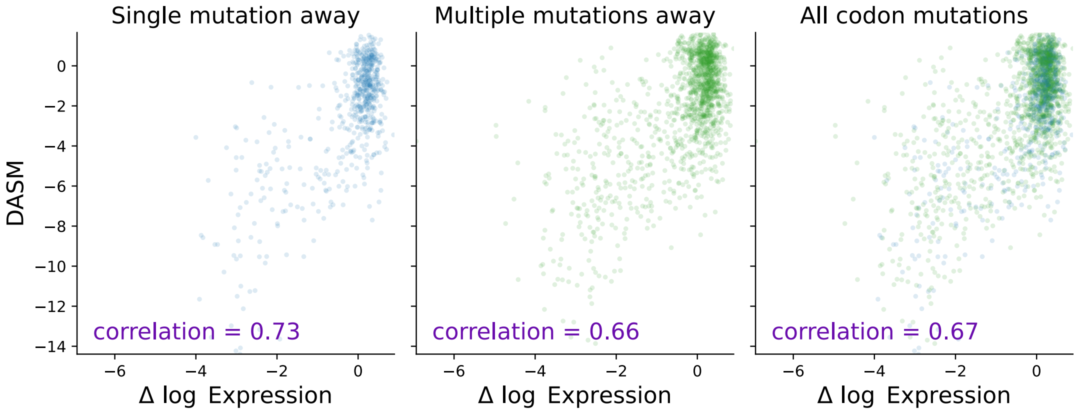

Figure S2: DASM removes codon bias in light chain functional predictions. Equivalent plot as Figure 3 but for light chain.

bioRxiv preprint doi: https://doi.org/10.1101/2025.10.21.683652; this version posted October 22, 2025. The copyright holder for this preprint (which was not certified by peer review) is the author/funder, who has granted bioRxiv a license to display the preprint in perpetuity. It is made available under aCC-BY 4.0 International license.

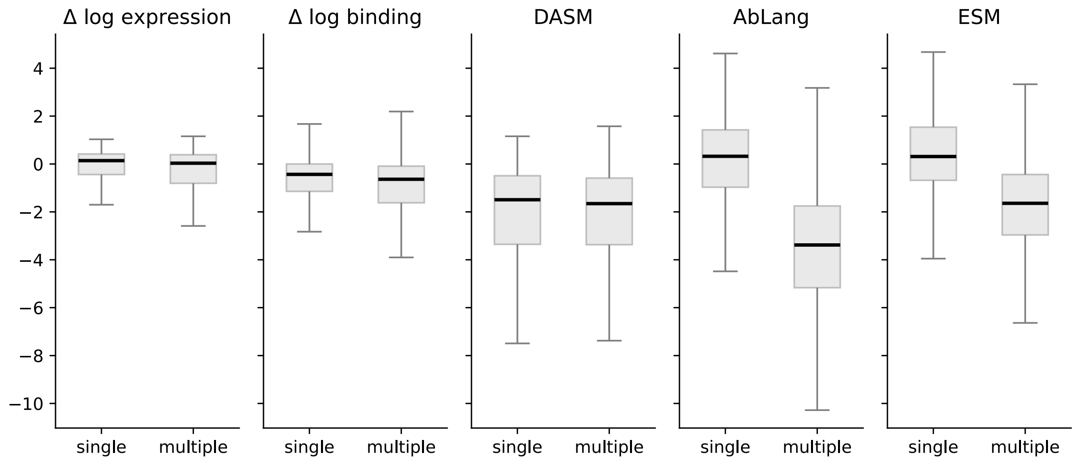

Figure S3: DASM selection factors are similar between codon neighbor amino acids and non-neighbors (compare Figure 1a), in contrast to other models. Comparison done on the heavy chain of the Koenig [11] wildtype sequence.

bioRxiv preprint doi: https://doi.org/10.1101/2025.10.21.683652; this version posted October 22, 2025. The copyright holder for this preprint (which was not certified by peer review) is the author/funder, who has granted bioRxiv a license to display the preprint in perpetuity. It is made available under aCC-BY 4.0 International license.

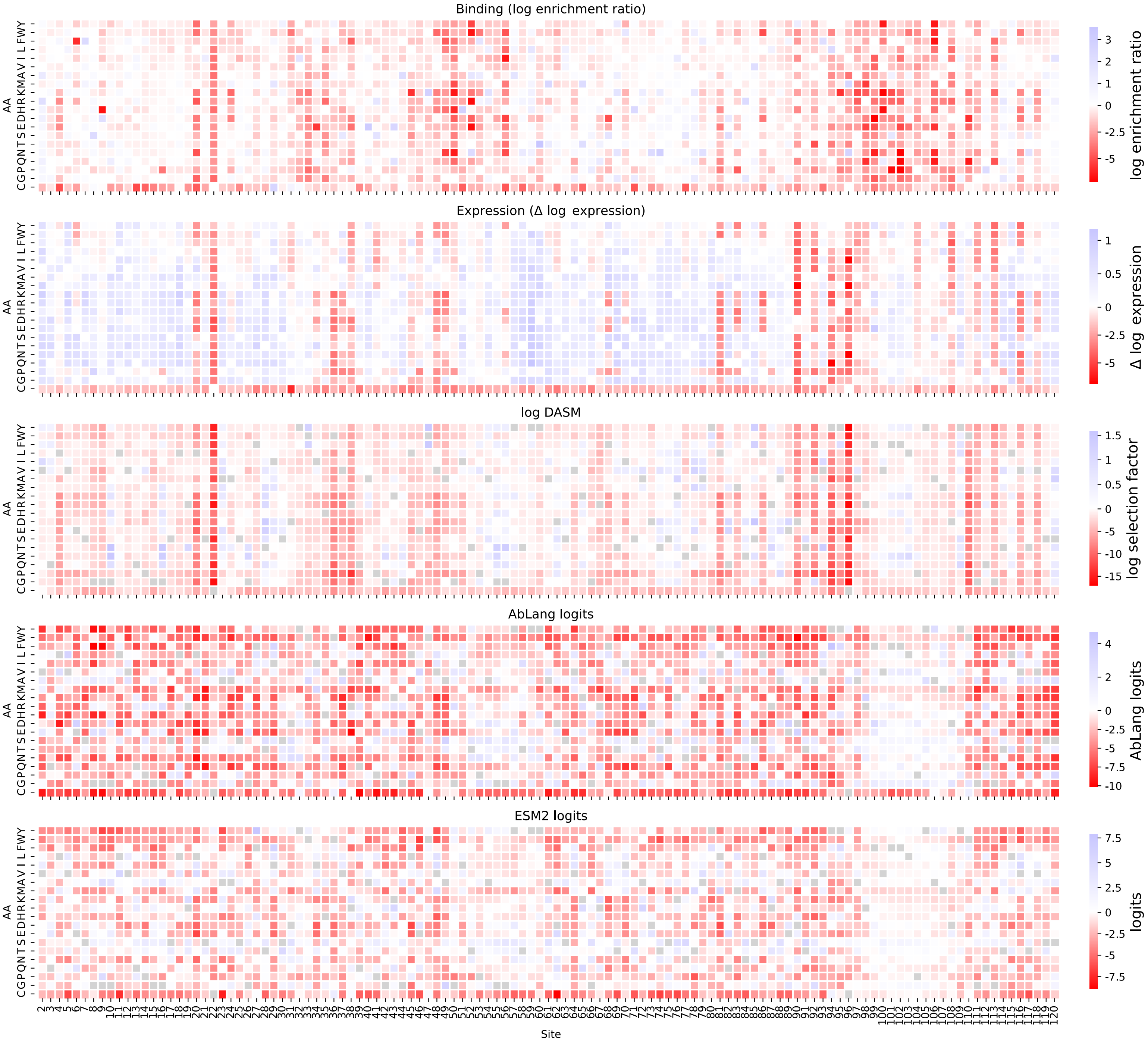

Figure S4: Heatmap showing the heavy-chain data of [11] along with model predictions.

bioRxiv preprint doi: https://doi.org/10.1101/2025.10.21.683652; this version posted October 22, 2025. The copyright holder for this preprint (which was not certified by peer review) is the author/funder, who has granted bioRxiv a license to display the preprint in perpetuity. It is made available under aCC-BY 4.0 International license.

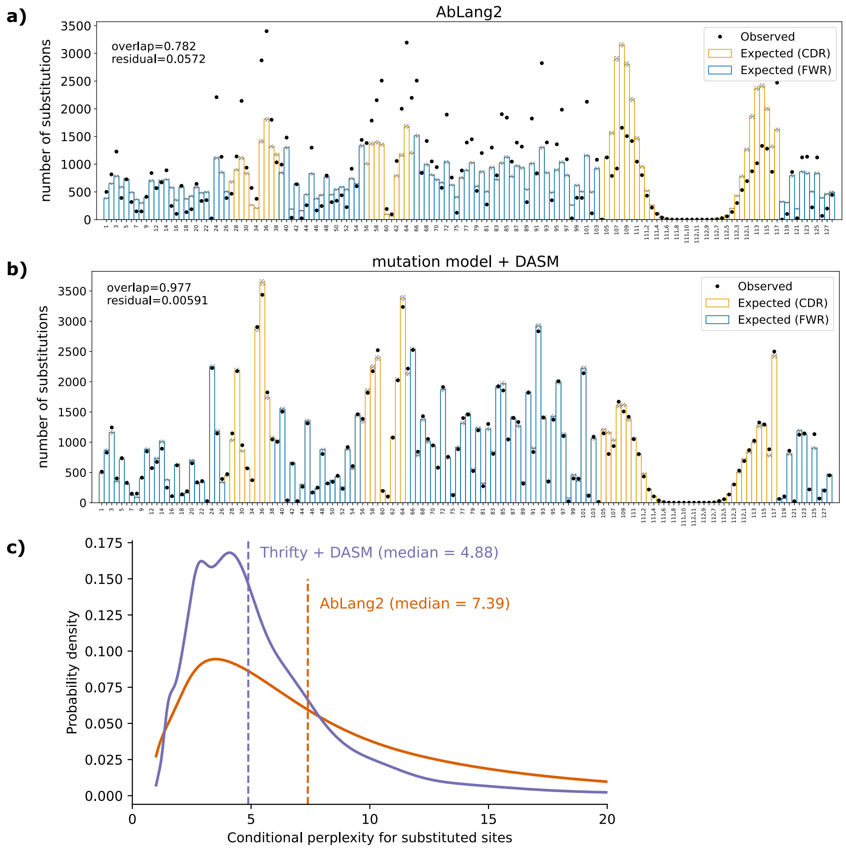

Figure S5: The DASM with the Thrifty neutral model accurately assesses probabilities of natural affinity maturation paths. **a**, **b**: The DASM is better than AbLang2 at predicting the location of nonsynonymous mutations observed in PCPs withheld from model training. **c**: The DASM achieves lower conditional perplexity (median 4.88 vs 7.39) when predicting amino acid identity at mutated sites, with fewer extreme outliers. The conditional perplexity is the perplexity of the child amino acid, conditioned on there being a mutation at that site. Note that due to the inherent stochasticity of affinity maturation, there is a lower limit to this conditional perplexity that is substantially greater than 1.

bioRxiv preprint doi: https://doi.org/10.1101/2025.10.21.683652; this version posted October 22, 2025. The copyright holder for this preprint (which was not certified by peer review) is the author/funder, who has granted bioRxiv a license to display the preprint in perpetuity. It is made available under aCC-BY 4.0 International license.

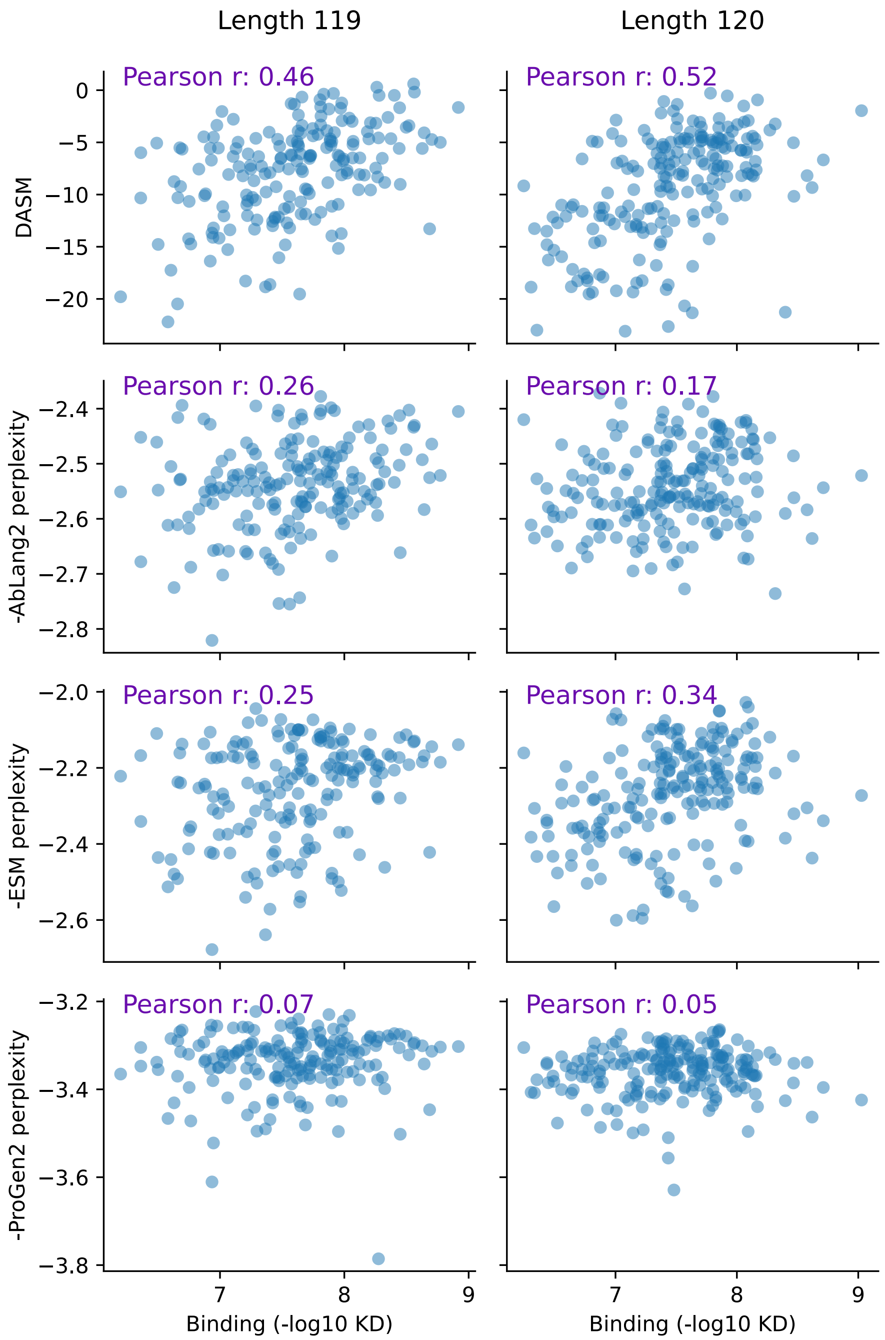

Figure S6: Scatterplots for the data of [23] zero-shot data set, partitioned by sequence length.

bioRxiv preprint doi: https://doi.org/10.1101/2025.10.21.683652; this version posted October 22, 2025. The copyright holder for this preprint (which was not certified by peer review) is the author/funder, who has granted bioRxiv a license to display the preprint in perpetuity. It is made available under aCC-BY 4.0 International license.

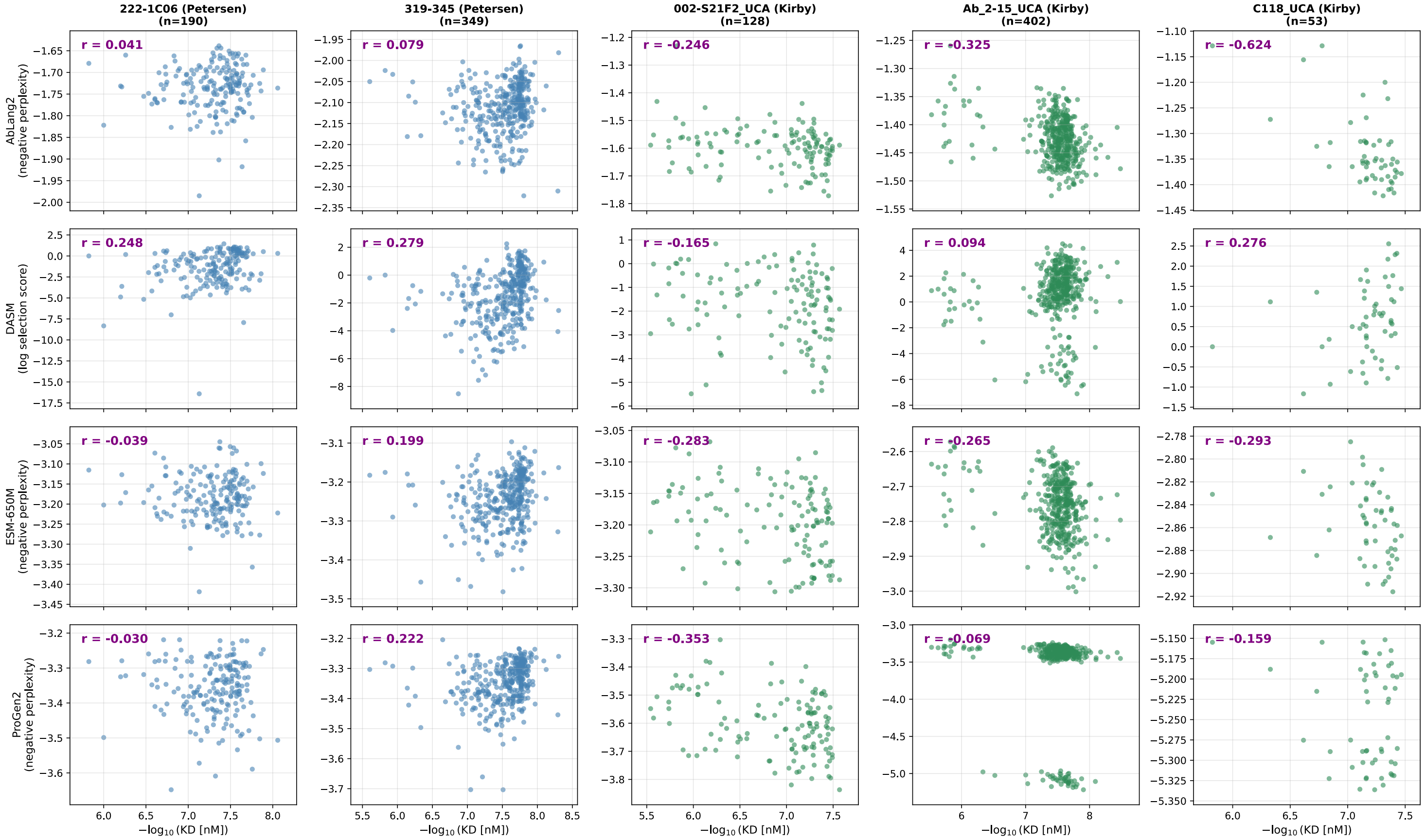

Figure S7: Model predictions versus experimentally measured binding affinity for five antibodies using the MAGMA-seq protocol. "Petersen" data are from an experiment probing the rules of recognition for influenza neutralizing antibodies [24] and "Kirby" data are from combinatorial libraries applying combinations of mutations on the path from naive to mature [26]. Each row shows a different model (DASM, ESM2, AbLang2, ProGen2) and each column shows a different antibody lineage.

bioRxiv preprint doi: https://doi.org/10.1101/2025.10.21.683652; this version posted October 22, 2025. The copyright holder for this preprint (which was not certified by peer review) is the author/funder, who has granted bioRxiv a license to display the preprint in perpetuity. It is made available under aCC-BY 4.0 International license.

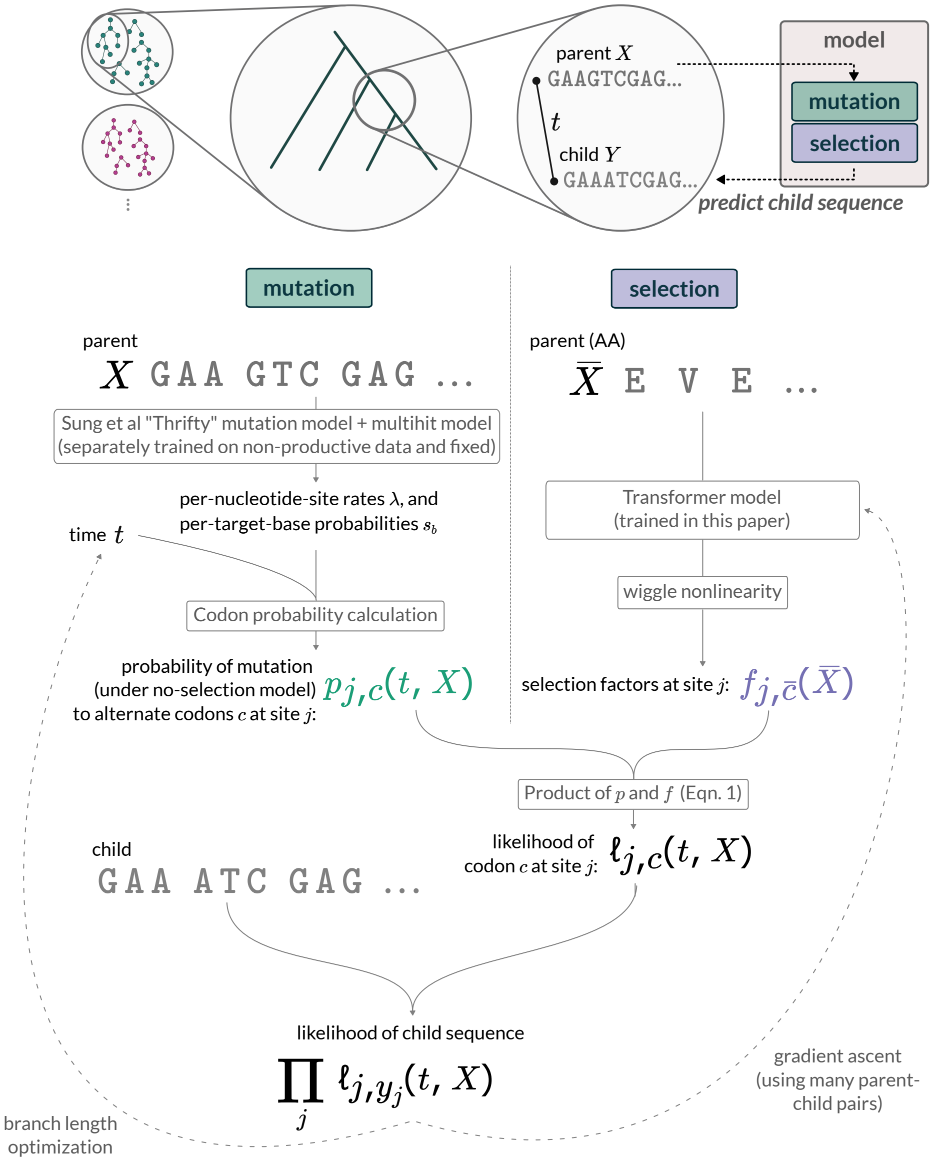

Figure S8: The model is trained to predict the probability of a child sequence given the parent sequence. It is divided into mutation and selection components. Mutation: given a parent sequence X, probability of mutation to alternate codons after time t is calculated using a model of SHM [7] and a "multihit model" (see Methods) and then aggregated into codons as in [15] to obtain $p_{j,c}(t, X)$. Selection: given an amino acid translation $\bar{X}$ of the parent, the transformer-encoder gives per-site selection factors $f_{j,\bar{c}}(\bar{X})$. These are then multiplied (1) and summed to give the probability of the observed child sequence at every site. This gives a likelihood for a parent-child pair. The algorithm maximizes the likelihood across branch lengths t for each parent-child pair as well as across the parameters of the transformer model for all parent-child pairs in the dataset (dashed lines).

bioRxiv preprint doi: https://doi.org/10.1101/2025.10.21.683652; this version posted October 22, 2025. The copyright holder for this preprint (which was not certified by peer review) is the author/funder, who has granted bioRxiv a license to display the preprint in perpetuity. It is made available under aCC-BY 4.0 International license.

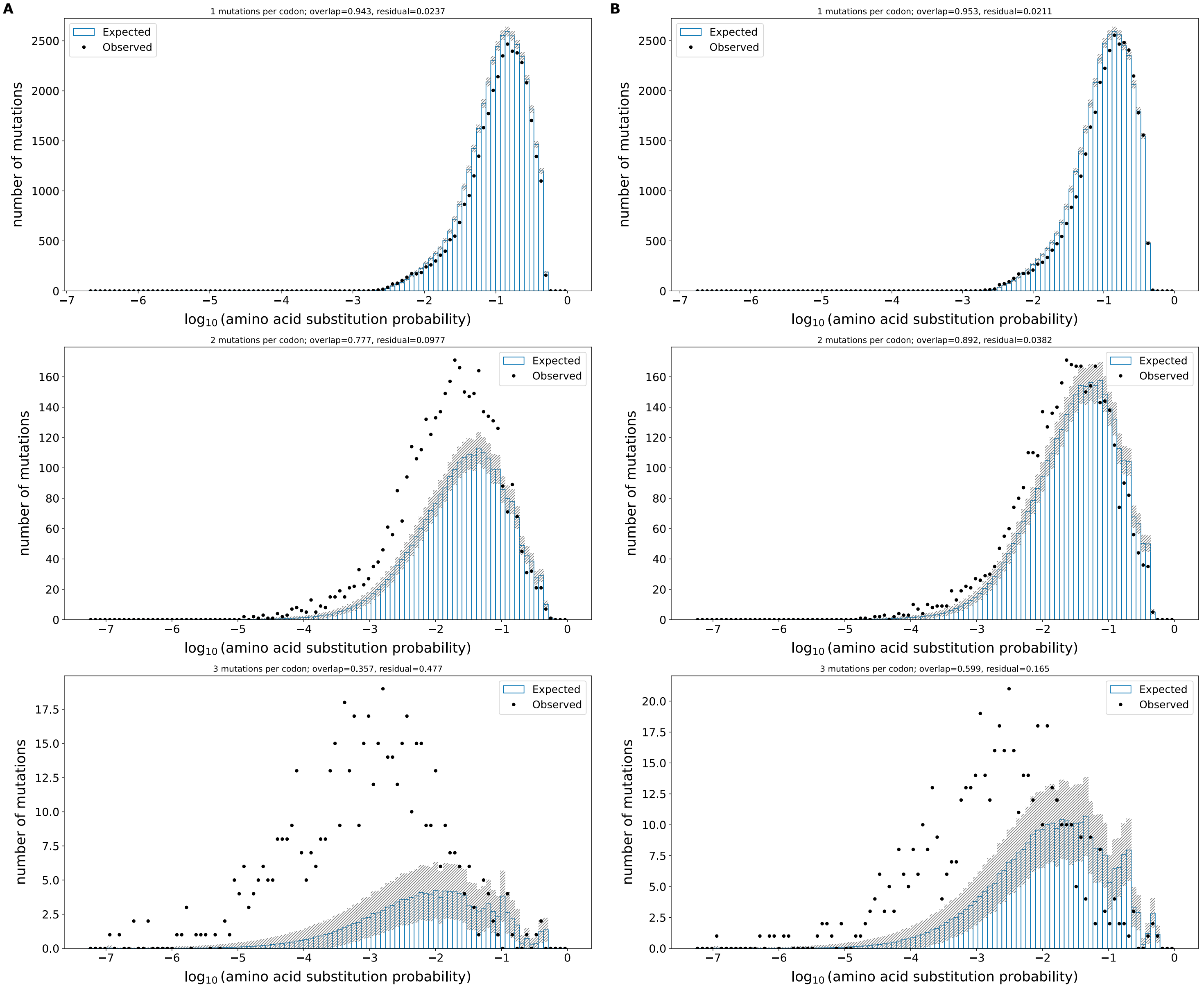

Figure S9: Models that allow for multiple hits per codon (right column) show better model fit than without (left column) on the out-of-frame data from [55] as prepared in [7]. These observed-versus-expected plots compare the observed number of mutations to the computationally predicted number across bins of mutation probability. For each bin, the expected number of mutations is calculated as the sum of mutation probabilities for all sites in that bin, while the observed count represents the actual mutations found in those sites. The overlap metric quantifies the area of overlap between observed and expected distributions divided by their average area, while the residual metric measures the normalized root-mean-square difference between observed and expected counts. These plots are faceted by the number of mutations per codon.
